# An *ab initio* study and machine learning framework to capture the motional effects in solid-state NMR of lithium-ion conductors

**DOI:** 10.1039/d6ta02026g

**Published:** 2026-06-02

**Authors:** Benjamin Zelin, Andrey D. Poletayev, Eleanor Davison, Gregory J. Rees, Bartholomew T. Payne, Peter G. Bruce, M. Saiful Islam, Jonathan R. Yates

**Affiliations:** a Department of Materials, University of Oxford Parks Road Oxford OX1 3PH UK benjamin.zelin@materials.ox.ac.uk saiful.islam@materials.ox.ac.uk jonathan.yates@materials.ox.ac.uk; b The Faraday Institution, Quad One, Harwell Science and Innovation Campus Didcot UK

## Abstract

Solid-state NMR spectroscopy, when combined with first-principles density functional theory (DFT) calculations, offers a highly sensitive probe of atomic-scale structure and dynamics in solid-state ion conductors, enabling the characterisation of subtle features that govern ionic conductivity. However, current approaches for interpreting NMR spectra rely on a comparison with static DFT reference calculations, which are inadequate for materials exhibiting fast ion dynamics such as lithium battery solid electrolytes. Here, using room-temperature NMR measurements and first-principles calculations, we show that the standard static-structure approach fails to reproduce the experimental ^35^Cl isotropic chemical shift (*δ*_iso_) of the fast Li-ion conductor Li_6_PS_5_Cl and substantially overestimates the quadrupolar coupling constant (*C*_Q_). We show that this discrepancy can be resolved using only ten DFT calculations by sampling relaxed configurations representative of Li-ion diffusion from machine-learning molecular dynamics. Compared with vibrational motion, Li-ion hopping around Cl is shown to dominate the motional averaging through reorientation of the NMR tensors. This study therefore provides an efficient computational method to resolve the complexities of the NMR spectra of Li_6_PS_5_Cl, which can be widely applied to other ion-conducting solids.

## Abstract 2

We present a novel methodology that combines machine-learning molecular dynamics with minimal DFT calculations to capture ion-motion effects in solid-state NMR. Applied to Li_6_PS_5_Cl, it provides a general framework for ion-conducting solids.

## Graphical abstract

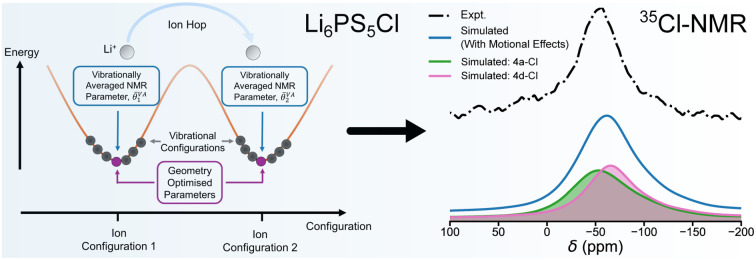

## 1 Introduction

**Fig. 1:**
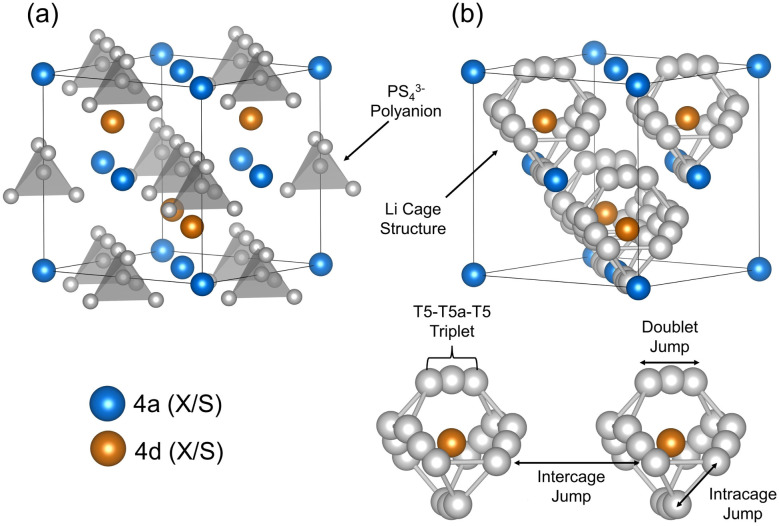
Generalised unit cell of Li_6_PS_5_X emphasising in (a) the positions of the PS_4_^3−^ units (grey polyhedra) and 4a/4d Wyckoff sites and in (b) the cage-like structures formed by the predominant Li ion sites.

Solid-state batteries that use lithium-ion conducting solid electrolytes are attracting considerable attention as safer and higher energy density alternatives to conventional lithium batteries that use flammable liquid electrolytes.^1–5^ Advances in solid electrolytes require materials design informed by a greater understanding of their properties on the atomic and nanoscale. Lithium argyrodites, Li_6_PS_5_X (X = Cl, Br and I), are among the most promising solid electrolytes, exhibiting ionic conductivities approaching 10 mS cm^−1^,^6–10^ suitable for high-performance solid-state batteries.^1,11^ For X = Cl and Br, partial anion disorder between the 4a and 4d Wyckoff sites (Fig. 1a) is observed, in contrast to the ordered iodide analogue, and this disorder is widely regarded as essential for achieving high ionic conductivity. Previous experimental^12–14^ and computational studies^15–18^ indicate that Li ions occupy cage-like environments surrounding the halide anions, with long-range transport requiring Li migration between adjacent cages (Fig. 1b). Anion disorder is thought to facilitate these intercage hops, thereby enhancing Li-ion conductivity.^10,19–21^ Despite this understanding, the role of atomic-scale structure and dynamics on the macroscopic conductivity remains incompletely understood.

A major challenge lies in characterising local Li environments in these disordered materials. Diffraction techniques are inherently limited by spatial averaging and poor sensitivity to short-range correlations.^22^ In contrast, solid-state NMR spectroscopy offers a unique sensitivity to subtle variations in local structure and dynamics. Variable-temperature NMR studies of lithium argyrodites using ^31^P and ^35^Cl as spectator nuclei have proven valuable for characterising the transport mechanisms.^23,24^ However, inconsistencies in the reported ^35^Cl spectra have led to conflicting peak assignments that hinder the systematic improvement of these materials highlighting the need for a more robust atomistic understanding of the structure and dynamics in these superionic conductors.^7–9,24,25^

**Fig. 2:**
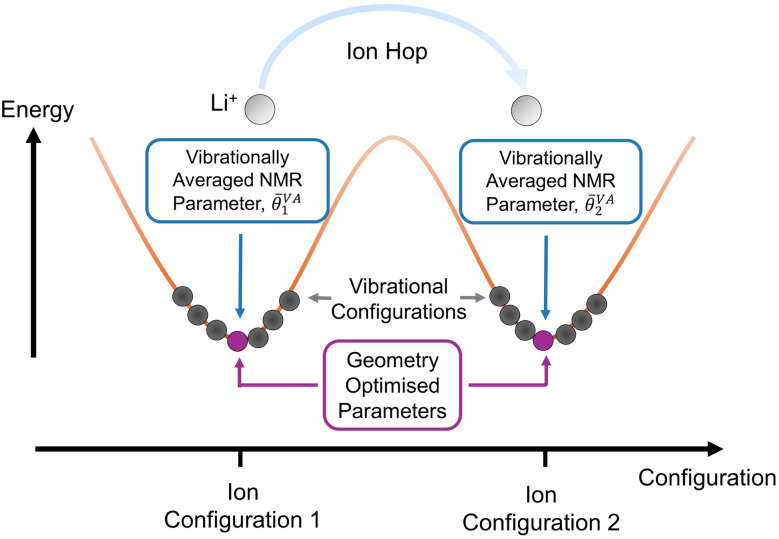
Schematic of the motional averaging effects of a general NMR parameter, *θ*, arising from two different timescales of lithium dynamics in solid-state conductors: vibrations within crystallographic sites and hopping between sites.

First-principles calculations provide a powerful framework for interpreting solid-state NMR spectra. By simulating the NMR interaction tensors for candidate structures, trends in peak positions that reflect subtle changes in the local atomic environments may be identified, and, ultimately, their impact on macroscopic ionic conductivity may be determined. However, such approaches typically rely on static reference structures, whereas ions in superionic conductors are highly dynamic at experimental temperatures. In lithium argyrodites, stochastic lithium motion and lattice vibrations lead to fluctuations of the local halide environments on timescales comparable to those probed by NMR (Fig. 2). These dynamics can result in significant averaging of NMR tensors over multiple local configurations, potentially giving rise to discrepancies between experimental and simulated spectra.

Simulating the influence of atomic motion on NMR spectra requires *ab initio* accuracy. Several first-principles approaches have been proposed, typically involving the generation of representative configurations and averaging the NMR tensors of a limited number of snapshots.^26^ Short-timescale vibrational effects can be treated using Monte Carlo sampling within the harmonic approximation^27–29^ or *via* perturbative expansions of the shielding tensor,^30^ while anharmonic motion can be captured using *ab initio* molecular dynamics (AIMD). AIMD cannot directly access dynamics on the timescale of NMR experiments (∼ns) and, moreover, insufficient sampling of correlated dynamics can bias ensemble-averaged NMR tensors.^31^ Classical molecular dynamics employing empirical interatomic potentials has been proposed to overcome these limitations; however, the accuracy of such potentials is often system-dependent, and consequently only limited improvements have been observed.^32,33^ Machine learning approaches for directly predicting the NMR tensors have also been proposed, but such models require an extensive DFT training set.^34–38^

Here we address these limitations by developing a first-principles framework to simulate the effects of motion on the ^35^Cl NMR spectra of Li_6_PS_5_Cl at room temperature. Our approach combines GIPAW^39–41^ calculations of chemical shielding and electric field gradient (EFG) tensors with a machine-learning interatomic potential (MLIP) to efficiently sample representative structures over NMR timescales. MLIPs reproduce the DFT potential energy surface with near-first-principles accuracy at a fraction of the computational cost,^42^ including for argyrodite ion conductors.^10,43^ To ensure unbiased sampling, we employ environment-based and statistical descriptors of the local Cl environments to guide snapshot selection and mitigate artefacts arising from time-correlated dynamics. Finally, by relaxing sampled configurations, we decouple short-timescale vibrational motion from slower Li-ion hopping, allowing us to isolate the microscopic origins of motional averaging in this material.

## 2 Results and discussion

### 2.1 Reproducing ionic conductivity

We validated the accuracy of our custom-trained MLIP in reproducing the atomic dynamics of Li_6_PS_5_Cl, as described in the SI. Because the dominant dynamical process influencing the local environments within the Cl cages is the hopping of neighbouring Li ions, we focused our validation on the ability of the MLIP to capture Li-ion hopping behaviour. This was done in two ways. First, we computed frequency-dependent ionic conductivities, *σ*(*ν*), and compared it with the bulk conductivity extracted from published impedance spectroscopy measurements (Fig. S4a).^44^ Secondly, we calculated the lithium self-diffusivity, D*, at several temperatures (Fig. S4b) to extract the activation energy associated with Li-ion diffusion. The extrapolated low-frequency limit of our simulated *σ*(*ν*) agrees well with the macroscopic conductivities previously measured.^44^ Furthermore, the activation energy for diffusivity obtained from our simulations (0.21 ± 0.01 eV) is in agreement with previous AIMD results (0.18 ± 0.02 eV).^19^ These comparisons demonstrate that our MLIP captures the essential Li-ion dynamics in Li_6_PS_5_Cl and is therefore sufficiently reliable for the analyses presented in this work.

### 2.2 Simulating the effect of ion motion on ^35^Cl NMR

To investigate the effect of motion on the ^35^Cl NMR parameters, we have performed 300 K *NPT*-MD simulations using our custom MLIP. Ideally, the chemical shift and EFG tensors would be averaged over every snapshot along the trajectory. However, the GIPAW calculations are computationally demanding, with each requiring approximately 2300 CPU hours when optimally parallelised.

**Fig. 3:**
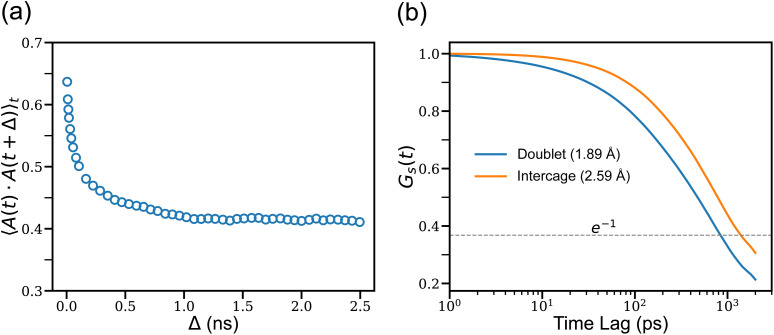
Timescales of the local motion of lithium ions. (a) Site–site correlation function for Cl cages over time. (b) Integral of the self part *G*_s_ of the van Hove correlation function up to distances corresponding to doublet and intercage jump displacements.

Since Li-ion hopping is expected to dominate the nanosecond dynamics of local environments within the Cl cages, our method focuses on sampling configurations representative of Li-ion hopping events. Furthermore, from our previous discussion uniformly spaced sampling along a trajectory can introduce uncertainty due to time-correlated dynamics, thereby biasing the ensemble averages. To mitigate this issue and to establish an appropriate sampling interval, we developed environment based descriptors, *A*, that quantitatively characterise the local Li-ion environments around each Cl atom. In short, each descriptor encodes which nearest neighbouring Li sites within a cage are occupied in Boolean vectors (Fig. S2). Fig. 3a shows the time correlation function of the environment descriptor. This function represents the timescale over which a given Cl environment remains correlated with itself and quantifies the persistence of a given local configuration. To complement this analysis, we have integrated the self part of the van Hove autocorrelation function (*G*_s_) for the Li ions over radii corresponding to displacements comparable with hopping distances. The decrease of this integral over time for a given radius estimates the probability of a Li ion being displaced by that distance over the same time.^45^ We computed this for displacements corresponding to two types of Li-ion hopping events, a hop within a given cage (doublet) and a hop between cages (intercage), both depicted schematically in Fig. 1. The latter jump is critical for long-range diffusion and is commonly considered the rate-limiting step in the ionic conduction process. A given hopping event can be judged to occur when *G*_s_ decays to approximately e^−1^. From the temporal decay of the integrated *G*_s_, we estimate that an intercage jump occurs on a timescale of approximately 0.5 ns. This timescale also corresponds to the decay of the Cl environment correlation function shown in Fig. 3a, indicating that local Cl environments remain correlated over roughly the same period. As such, optimal sampling should be able to omit structures that are less than 0.5 ns apart without loss of accuracy.

**Fig. 4:**
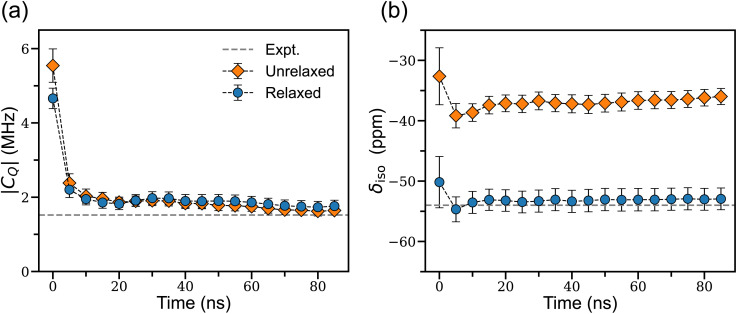
Long-timescale averaged ^35^Cl NMR parameters. (a) Convergence of the simulated quadrupolar coupling constant towards experimental values (dashed) using unrelaxed (orange diamonds) and relaxed (blue circles) structures. (b) Convergence of the simulated chemical shifts using unrelaxed (orange diamonds) and relaxed (blue circles) structures.

**Fig. 5:**
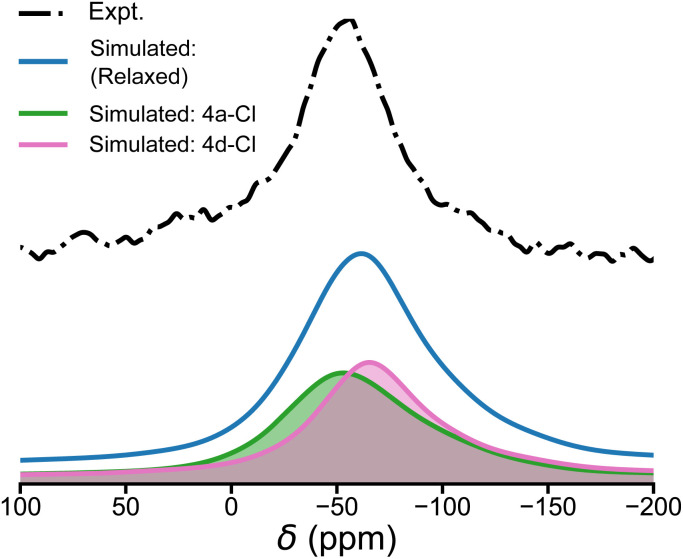
Comparison of the experimental static ^35^Cl NMR spectrum of Li_6_PS_5_Cl with the spectrum simulated using our first-principles framework. The simulated spectrum is further decomposed into contributions from Cl ions occupying the 4d and 4a crystallographic sites.

The two parameters that most strongly determine the shape and position of the ^35^Cl NMR spectrum in Fig. 5 are the quadrupolar coupling constant, |*C*_Q_|, which primarily controls the spectral width, and the isotropic chemical shift, *δ*_iso_, which determines the peak position. By averaging the full chemical shift and EFG tensors at snapshots sampled at every 0.5 ns we have computed the time-convergence series for *C*_Q_ and *δ*_iso_ respectively, in Fig. 4a and b. To decouple the effects of rapid vibrations, each sampled structure was geometry optimised (termed “relaxed” below) to its local potential-energy minimum. As demonstrated schematically in Fig. 2, this procedure quenches the high-frequency vibrational contributions and isolates the influence of slower dynamics, such as Li-ion hopping, on the NMR parameters. For comparison, results obtained from snapshots taken directly from the MD trajectory (termed “unrelaxed” below) are also presented to assess the effect of geometry relaxation on the predicted ^35^Cl NMR spectra.

Focusing on the quadrupolar coupling (Fig. 4a), the static structure at *t* = 0 yields a |*C*_Q_| value of 4.66 ± 0.28 MHz, with the error bar representing the distinct Cl atoms in the computed structure. This is significantly higher than the experimentally derived 1.52 ± 0.05 MHz. This indicates that a single static calculation substantially overestimates the peak width of the ^35^Cl NMR spectra in Li_6_PS_5_Cl, preventing meaningful conclusions from being drawn from trends in such static results. In contrast, using our relaxed snapshots method, we obtain an averaged value of 1.76 MHz, converged to within ±0.02 MHz after 68 snapshots—showing much better agreement with experiment. This significantly improved agreement verifies that diffusive Li-ion hopping is the dominant contributor to the motional averaging observed in the experimental value of |*C*_Q_|. Similarly, for the unrelaxed structures, the computed |*C*_Q_| converges to 1.66 MHz (within ±0.03 MHz) after 69 snapshots, also in excellent agreement with experiment. This result indicates that short-timescale local dynamics have a negligible influence on the motional averaging of the electric field gradient (EFG) tensor compared with the longer-timescale changes in Cl environments induced by Li-ion diffusive motion.

Fig. 4b shows the time-convergence of the isotropic chemical shift, *δ*_iso_. Unlike for |*C*_Q_| the relaxed static structure at *t* = 0 already yields an isotropic chemical shift of −50.17 ± 4.25 ppm, in much closer agreement with the experimental value of −54.01 ± 0.17 ppm. After approximately 60 snapshots, *δ*_iso_ converges to −52.9 ppm, stable within ±0.1 ppm. This convergence timescale is comparable to that observed for |*C*_Q_|. In contrast to |*C*_Q_|, however, the time-averaged value from unrelaxed snapshots differs significantly from those of the relaxed snapshots. The unrelaxed static structure exhibits an initial *δ*_iso_ of −32.64 ± 4.72 ppm, which converges to −32.02 ± 1.31 ppm after 87 snapshots. Although the convergence trend is qualitatively similar to that of the relaxed structures, the substantial offset indicates a systematic difference in how *δ*_iso_ is derived from the time-averaged shielding tensor.

As described in the SI, *δ*_iso_ is obtained from the isotropic magnetic shielding, *σ*_iso_, according to

1 *δ*_iso_ = *σ*_iso_ − *σ*_ref_,

where *σ*_ref_ is a reference shielding constant linking the DFT-calculated *σ*_iso_ to the experimental *δ*_iso_. Since *σ*_ref_ cannot easily be computed from first-principles, it is typically determined *via* linear regression between DFT-calculated *σ*_iso_ and experimental *δ*_iso_ values for ^35^Cl (Fig. S3). In this work, the reference shielding values were derived using relaxed structures. We therefore propose that the systematic offset between the relaxed and unrelaxed convergence series arises from this choice of reference. Specifically, the relaxed structures, in which vibrational motion has been quenched, inherently include a systematic correction for vibrational averaging that is absent in the unrelaxed configurations. Furthermore, the similarity of this correction across both the reference compounds and the lithium argyrodite system implies a consistent influence of vibrational averaging on the computed *δ*_iso_ values. Further work will be required to confirm this interpretation.

Whilst the fluctuations in *C*_Q_ and *δ*_iso_ become negligible after roughly 60–70 snapshots, the convergence rate is more clearly assessed by examining the evolution of the error bars in Fig. 4a and b. These error bars represent the standard deviation of the time-averaged parameters across the different Cl sites in the simulation cell (Fig. S1). At the beginning of the simulation, the large error bars reflect the lack of convergence with respect to time. However, after only 9–10 snapshots, the error bars decrease and stabilise to within 0.01 MHz for |*C*_Q_| and 0.1 ppm for *δ*_iso_, indicating that both parameters have effectively converged. The remaining variance is attributed to differences in second-nearest-neighbour environments and their dynamics rather than to Li-ion motion. This finding demonstrates that time-converged parameters can be obtained with as few as 10 snapshots. We note it is also possible to simulate the effects of dynamics by machine-learning the full NMR tensors. For disordered systems such as Li_6_PS_5_Cl, however, this approach would necessitate performing a large number of DFT calculations on sufficiently large simulation cells. A similarly large number of DFT calculations has also been required in previous studies that sampled MD trajectories. The advantage of our method is that compared to these studies the number of DFT calculations required to achieve convergence are relatively small, therefore, the primary computational cost shifts away from the DFT calculations and towards the training of the MLIP. In this regard, recent advances in universal MLIPs and the potential for their inexpensive fine-tuning at the appropriate level of theory^42,46^ promise to reduce the cost of MLIP training substantially. Our method therefore offers a viable and efficient route for incorporating motional effects in future NMR analyses of ion conductors.

For a qualitative judgement of our method, we have also simulated in Fig. 5 the ^35^Cl NMR spectrum using our motionally averaged tensors and compared it with the experimental spectra (see SI for simulation and experimental details). Relative to the spectra simulated for a single static structure (Fig. S5), the motionally averaged spectrum shows excellent agreement with experiment, with both the peak position and overall lineshape closely reproduced. These results highlight the importance of properly accounting for motional averaging in order to accurately interpret the ^35^Cl NMR spectra of Li_6_PS_5_Cl. The figure also separates the individual contributions from Cl atoms on the 4a and 4d Wyckoff sites. Our motional-averaging method reveals that these two sites give rise to statistically distinguishable peaks, even though they are not resolvable in the experimental spectrum. In the experimental ^35^Cl NMR spectra of Li_6_PS_5_Cl, the observation of a third distinct resonance has previously been reported. For instance, Feng *et al.*^9^ identified peaks at 9 and −25 ppm, which were attributed to Cl^−^ ions residing on the 4d and 4a crystallographic sites, respectively. Our results confirm that the peak at 9 ppm does not originate from Cl atoms occupying a distinct Wyckoff site, but is instead more likely associated with a secondary phase. We further confirmed that this peak arises from heating the material above 250 °C (Fig. S6) and is consistent with the appearance of LiCl as a product of thermal decomposition.^47^

We note that the influence of disorder on the 4a/4d Wyckoff sites, while not directly investigated here, is of critical importance in optimising the ionic conductivities of argyrodite based solid electrolytes.^18,48^ Our simulations indicate that differences in the peak positions corresponding to 4a and 4d sites fall below the threshold of experimental resolution (Fig. 5). Consequently, we expect that changes in the degree of disorder will have a negligible impact on the ^35^Cl NMR chemical shift. The primary influence of disorder is instead anticipated in the evolution of the peak shape, driven by secondary effects related to altered ion diffusivity.

### 2.3 Atomistic insights into motional averaging

**Fig. 6:**
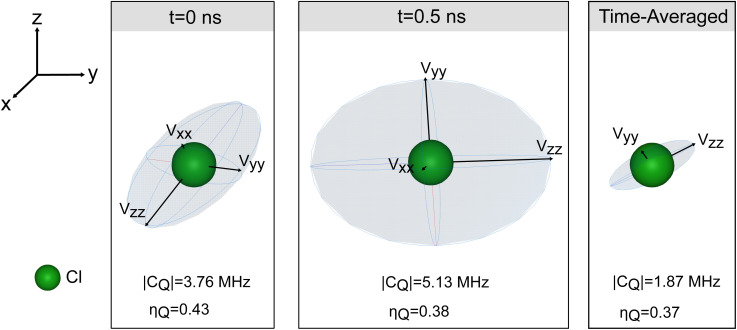
Visualisation of static electric field gradients of a selected ^35^Cl of Li_6_PS_5_Cl at two subsequent uncorrelated timesteps compared with its time-averaged tensor. Quadrupolar coupling constants, |*C*_Q_|, asymmetry parameters *η*_Q_ and eigenvectors *V*_*ii*_ are highlighted (see SI for definitions).

To understand why our method so effectively reproduces the experimental spectra, it is useful to consider the microscopic mechanisms by which motion influences the NMR tensors. As Dračínský and Hodgkinson^49^ point out, motional averaging can arise from two different effects. First, a Li-ion hop that changes Li–Cl distances instantaneously alters the electric field gradient and, consequently, the tensor magnitudes. Second, because the tensors can rotate during motion, averaging over these different orientations can yield more isotropic tensors (Fig. 6). Following a similar analysis, we characterise the effect of atomic motion on tensor anisotropy by computing the reduced chemical shift anisotropy, *δ*_aniso_, and the quadrupolar coupling constant, *C*_Q_ (see SI for definitions), in three different ways, in Table 1. We first evaluate these quantities for a single static snapshot at *t* = 0 (*δ*^(static)^_aniso_ and *C*^(static)^_Q_) and for the time-averaged tensors obtained from our previous trajectory (*δ*^(avg.)^_aniso_ and *C*^(avg.)^_Q_). We then compute the values for each snapshot independently and average them (*δ*^(ind.)^_aniso_ and *C*^(ind.)^_Q_). The latter approach isolates the effect of tensor reorientation from the motional averaging process, allowing us to estimate its contribution by comparing (i) the difference between the static and time-averaged parameters with (ii) the difference between the static and independently averaged parameters. For both parameters the full reduction in anisotropy cannot be accounted for by the static averaging method. This result suggests that tensor reorientation is the dominant mechanism of motional averaging in this system. Furthermore in the case of the EFG tensor almost the entire reduction in anisotropy can be accounted for by tensor reorientation. To illustrate this mechanism, Fig. 6 compares the EFG tensors at two consecutive snapshots with the time-averaged tensor. The tensors at independent snapshots show significant reorientation and large anisotropies, whereas averaging these reoriented tensors over time yields a much more isotropic tensor with a substantially reduced magnitude. During Li-ion diffusion the Cl ions therefore sample a more uniform, time-averaged charge distribution.

**Table 1:** Calculated ^35^Cl NMR tensor anisotropy parameters of Li_6_PS_5_Cl. Superscript static refers to a single static structure, ind. indicates parameters independently averaged over MD snapshots, and avg. denotes values obtained by averaging the full NMR tensors for each MD snapshot. *β* represents the average reorientation angle of the magnetic shielding (MS) and electric field gradient (EFG) tensor principal components relative to a reference orientation due to Li diffusion

*δ* ^(static)^_aniso_ (ppm)	*δ* ^(ind.)^_aniso_ (ppm)	*δ* ^(avg.)^_aniso_ (ppm)	*β*_MS_ (°)	*C* ^(static)^_Q_ (MHz)	*C* ^(ind.)^_Q_ (MHz)	*C* ^(avg.)^_Q_ (MHz)	*β*_EFG_ (°)
5.78 (±5.78)	3.85 (±1.51)	1.52 (±1.96)	85.08 (±5.72)	4.65 (±0.28)	4.30 (±0.03)	1.76 (±0.16)	89.6 (±5.18)

To further compare our results with those of Dračínský and Hodgkinson,^49^ we computed the eigenvectors of the magnetic-shielding and EFG tensors at each timestep of our trajectory and evaluated the projection angle, *β*, between the principal components and a reference frame defined by the eigenvectors of the static structure at *t* = 0. The resulting quantities, *β*_MS_ and *β*_EFG_, therefore represent the average tensor-reorientation angles. Dračínský and Hodgkinson^49^ reported average reorientation angles of approximately 4°–14° for the shielding tensors in their peptide systems. In contrast, we observe substantially larger reorientation angles, reflecting the significant motional-averaging effects induced by tensor reorientation during Li-ion hopping. We therefore attribute the pronounced motional averaging in Li-ion conducting systems primarily to the substantial tensor reorientations that accompany Li-ion migration.

**Fig. 7:**
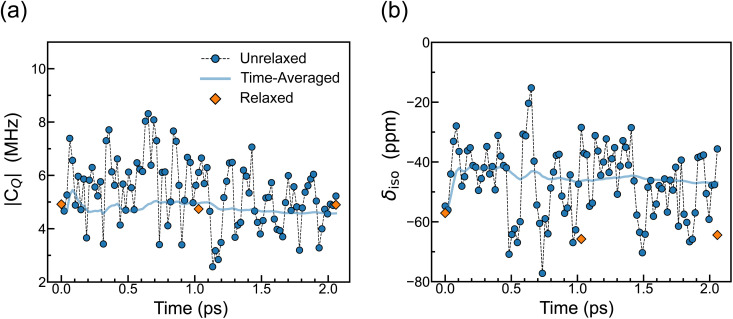
Short-timescale averaging NMR parameters for one selected ^35^Cl of Li_6_PS_5_Cl, |*C*_Q_| (a), and *δ*_iso_ (b), obtained from MD snapshots sampled at twice the frequency of the highest-energy vibrational mode in (a). The time-averaged values (blue line) are also plotted along with the effect of geometry relaxation for three snapshots sampled from this time series (orange diamonds).

The excellent agreement between the experimental spectra and those simulated using our sampling method suggests that motional averaging is dominated by the diffusion of Li ions around Cl on nanosecond timescales. To verify this, we explicitly examined the influence of Cl and Li vibrations by sampling configurations representative of their vibrational motion. According to the Nyquist theorem, capturing vibrational snapshots requires sampling at twice the frequency of the highest relevant vibrational mode. To estimate this frequency, we computed the velocity autocorrelation function from a 300 K *NPT* simulation (Fig. S7), whose Fourier transform reflects the vibrational density of states (vDOS). Decomposing the vDOS into elemental contributions shows that Cl vibrations occur below 10 THz, whereas Li vibrations extend up to 20 THz. We therefore sampled configurations at 20 THz to represent both species adequately. Fig. 7a and b show the resulting instantaneous (“unrelaxed”) values of *C*_Q_ and *δ*_iso_, together with their time-averaged (“time-averaged”) behaviour for one representative Cl atom. Vibrational motion produces large instantaneous fluctuations, comparable in magnitude to the averaged values. However, these fluctuations have minimal impact on the time-averaged quantities, which converge qualitatively within only 2–3 snapshots. We therefore propose that vibrational motion does not significantly contribute to the motional averaging effect. This is consistent with the weak influence on the convergence behaviour between relaxed and unrelaxed snapshots in Fig. 4.

To further verify this interpretation, we selected three snapshots and fully relaxed them (“relaxed”). For *C*_Q_, relaxation returns the values to the time-averaged limit. For *δ*_iso_, relaxation introduces a small offset (∼20 ppm) relative to the time-averaged value, which is identical to that observed in Fig. 4b. If this systematic shift is attributed to artefacts in the reference shielding, then the relaxed and time-averaged values are also consistent. Taken together, these results suggest that the vibrationally averaged NMR parameters reflect those of the local ground-state configuration about which the atoms vibrate (Fig. 2). Vibrations therefore introduce only random noise that averages out to zero, explaining why our original long-timescale sampling reproduces experiment without explicitly accounting for short-timescale vibrational dynamics. These observations indicate that vibrational motion does not contribute significantly to the motional averaging of the NMR parameters. Instead, the dominant mechanism is the long-timescale diffusion of Li ions.

## 3 Summary and conclusions

This work demonstrates a computationally efficient first-principles machine-learning based methodology for capturing motional averaging effects in the NMR spectra of the Li-ion solid electrolyte Li_6_PS_5_Cl. The methodology is highly applicable to other ion-conducting solids, where motional averaging is dominated by ion diffusion. Based on the present work, we propose the following sampling protocol: first, an interatomic potential must be selected that accurately captures ion dynamics, as demonstrated here using machine learning potentials for Li_6_PS_5_Cl. DFT-NMR calculations are then performed on MD snapshots sampled over the characteristic ion diffusion timescales to obtain time-averaged NMR parameters. We identified these sampling timescales using position-based autocorrelation functions – for the system studied here, this timescale was approximately 0.5 ns. We demonstrate that such an approach not only generates snapshots representative of the diffusion dynamics, but also ensures the local environments sampled are not biased by time-correlated dynamics.

Applying this method to the ^35^Cl NMR spectra of Li_6_PS_5_Cl we draw the following conclusions:

(a) Evenly spaced snapshots from long MD trajectories exhibit time-correlation bias in the local Li configurations around Cl. This bias is removed when sampling over timescales corresponding to the intercage jump process, which effectively destroys the time correlation.

(b) Conventional static first-principles calculations fail to reproduce the experimental isotropic chemical shift, *δ*_iso_, and significantly overestimate the quadrupolar coupling constant, *C*_Q_, of ^35^Cl. By contrast, averaging over a relatively small number of relaxed snapshots (∼10) representative of Li-ion diffusion reproduces both *δ*_iso_ and *C*_Q_ with near-experimental precision. Therefore, the developed method offers a computationally efficient framework for future applications.

(c) The primary source of motional averaging is the reorientation of the magnetic shielding and electric field gradient tensors as Li ions diffuse. For *C*_Q_, this tensor reorientation accounts for nearly the entire reduction in magnitude. The substantial decrease in *C*_Q_ indicates that Li-ion diffusion causes Cl atoms to experience on average a more uniform charge density.

(d) Averaging over unrelaxed snapshots has no effect on the convergence of *C*_Q_ compared to sampling relaxed snapshots. For *δ*_iso_, the time-convergence behaviour is qualitatively similar, but a systematic offset appears. We attribute this offset to inconsistent reference shielding values, which are computed using relaxed structures.

(e) Vibrational averaging has minimal effect on either *C*_Q_ or *δ*_iso_. The experimentally observed NMR parameters are therefore dominated by the configurational changes associated with Li-ion diffusion, rather than by short-timescale vibrational dynamics.

## Author contributions

Benjamin Zelin: conceptualization, methodology, software, data curation, investigation, validation, formal analysis, visualization, project administration, writing – original draft, and writing – review and editing; Andrey D. Poletayev: methodology, validation, software, supervision, writing – original draft, and writing – review and editing; Eleanor Davison: conceptualization; Gregory J. Rees: data curation, validation, writing – original draft, and writing – review and editing; Bartholomew T. Payne: data curation; Peter G. Bruce: resources; M. Saiful Islam: resources, supervision, funding acquisition, and writing – review and editing; Jonathan R. Yates: supervision, resources, project administration, funding acquisition, software, writing – original draft, and writing – review and editing.

## Conflicts of interest

The authors declare no competing financial interests or personal relationships that could have appeared to influence the work reported in this paper.

## Supplementary Material

TA-014-D6TA02026G-s001

## Data Availability

Raw CASTEP input files and output (magres) files are available in a Zenodo repository at https://doi.org/10.5281/zenodo.18913644. The machine learning interatomic potential is hosted in a separate repository that has not yet been published at the time of writing as it forms part of a larger ongoing study by the group but will be hosted at https://doi.org/10.5281/zenodo.18902499. The data supporting this article have been included as part of the supplementary information (SI). Supplementary information: computational and experimental methodology, ionic conductivities, static simulated NMR spectra, experimental temperature-dependent NMR spectra, vibrational density of states. Predicted ionic conductivities obtained using the machine learning interatomic potential (Fig. S4); first-principles simulations of static ^35^Cl NMR spectra (Fig. S5); experimental temperature-dependent ^35^Cl NMR spectra (Fig. S6); and vibrational densities of states predicted by the machine learning interatomic potential (Fig. S7). See DOI: https://doi.org/10.1039/d6ta02026g.
