# Supplementary material for: An ab initio study and machine learning framework to capture the motional effects in solid-state NMR of lithium-ion conductors
Source: J Mater Chem A Mater. 2026 Jun 2;14(41):28000–8. doi: 10.1039/d6ta02026g (PMC13249006; doi:10.1039/d6ta02026g)
Supplement: TA-014-D6TA02026G-s001 [file TA-014-D6TA02026G-s001.pdf]

Supplementary Information (SI) for Journal of Materials Chemistry A.
This journal is © The Royal Society of Chemistry 2026

An Ab Initio Study and Machine Learning Framework to Capture the Motional Effects in Solid-State NMR of Lithium-Ion Conductors

Benjamin Zelin,[1] Andrey D. Poletayev,[1,2] M. Saiful Islam,[1,2] Bartholomew T. Payne,[1,2] Peter G. Bruce,[1,2] and Jonathan R. Yates[1]

[1] *Department of Materials, University of Oxford, Oxford, OX1 3PH, United Kingdom*

[2] *The Faraday Institution, Quad One, Harwell Science and Innovation Campus, Didcot, OX11 0RA, United Kingdom*

(Dated: May 20, 2026)

METHODS

A. MLIP Architecture and Training

To train a machine-learning interatomic potential ground-truth density-functional theory calculations were carried out in VASP using the projector-augmented wave method [1, 2] with the PBEsol exchange-correlation functional [3]. The energy cut-offs were 600 eV and k-point density 0.25 $Å^{-1}$. Ab initio molecular dynamics were carried out at 300 K to 1200 K with a Langevin thermostat in isovolumetric (NVT) and isobaric (NPT) ensembles for simulation cells containing one conventional unit cell of $Li_5PS_4Cll_2$, Li_6PS_5Cl, and Li_7PS_6. Li_6PS_5Cl AIMD was sampled with 0%, 50%, and 100% S occupancy on 4d Wyckoff sites. Enhanced sampling of cell coordinates (if isobaric) and lithium diffusion via pairwise distances (if isovolumetric) was used via simple metadynamics biasing in VASP. The energy convergence was 10^{-6} eV with an 'Accurate' precision setting and time step 5 fs. The AIMD frames were used directly to train the *allegro* interatomic potentials [4, 5] from scratch. The cut-off radius was 5.0 Å, polynomial basis set of Bessel functions with cut-off 6, and angular number $l_{max} = 1$. the leading-edge embedding multiplicity for tensorial features was 32. The latent multi-layer perceptron dimensions were 256×3 layers with a silu nonlinearity. The two-body latent potential had 4 layers with dimensions 64, 128, 256, and 256. The final pairwise potential had a dimension of 128. The package versions are nequip 0.6.0 [4], allegro 0.2.0 [5], and e3nn 0.5.1 [6]. The potential was trained for 221 epochs at a learning rate of 0.002 (12 hours on one NVIDIA V100 GPU) with the Adam optimizer [7] and a random 70%-15%-15% train-validation-test split. The trained model, its complete training configuration and trace, and the full dataset of 28100 frames are available at ref [8].

We include for illustrative and learning purposes the distributions of energies and densities (Figure 1), and lattice parameters arising from enhanced sampling in the isobaric ensemble (Figure 2), in the collected training dataset. Note that the horizontal lines in Figure 2 are the result of simulations in the isovolumetric ensemble. The errors in energies, force magnitudes, and force direction are included in Figure 3 following best practices [9]. There are no systematic and apparent shifts or correlations in the errors.

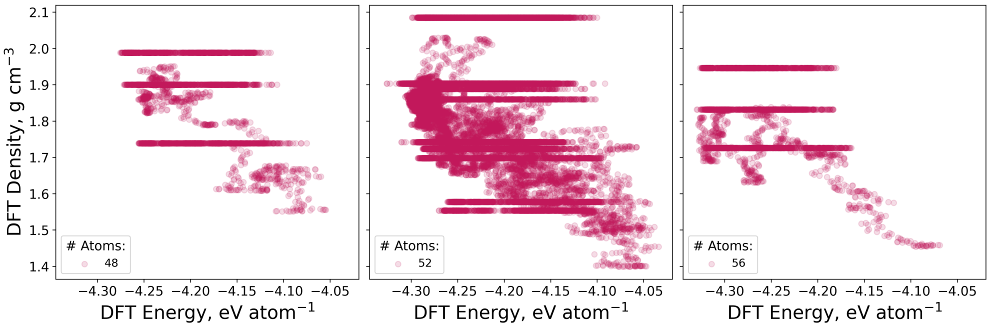

FIG. 1. Distributions of densities and energies for the DFT training data. The numbers of atoms in the panels correspond to three stoichiometries, viz. $Li_5PS_4Cl_2$ for 48 atoms, Li_6PS_5Cl for 52 atoms, and L_7PS_6 for 56 atoms. The horizontal lines correspond to simulations in the isovolumetric ensemble, where the energy per atom changes with temperature. The range of energies sampled with AIMD corresponds to simulations between 300 K to 1200 K.

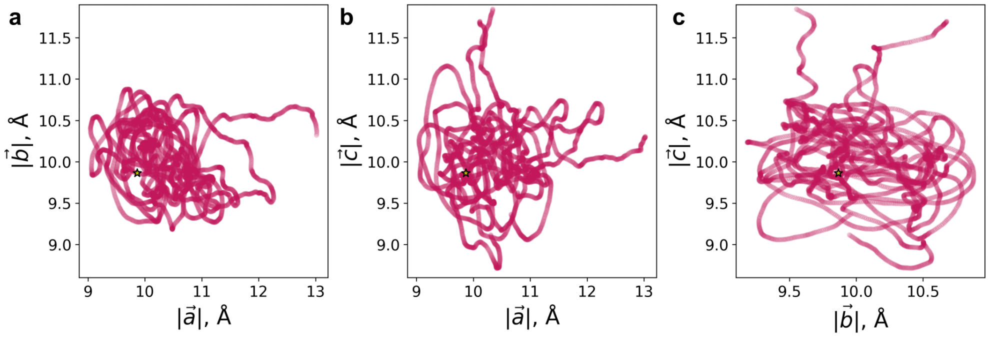

FIG. 2. Sampling of lattice parameters in isobaric simulations. The lattice parameters are shown for (a) ab-plane, (b) ac-plane, and (c) bc-plane. The experimental lattice constants of Li_6PS_5Cl are denoted with the yellow star in each panel.

B. Molecular Dynamics

All MD simulations were performed using the LAMMPS code [10]. To construct the simulation cell, an anion ordered conventional unit cell was first created with initial Li positions arranged octahedrally around the 4d Wyckoff site (Figure 4). $2 \times 2 \times 1$ supercells were created by repeating the conventional unit cell twice along the x and y axis. This supercell size was chosen to allow the DFT calculations to be performed with reasonable computational expense, whilst capturing the disorder in the material. Finally, to model the anion disorder, half of the S atoms on the 4d Wyckoff sites were randomly swapped with

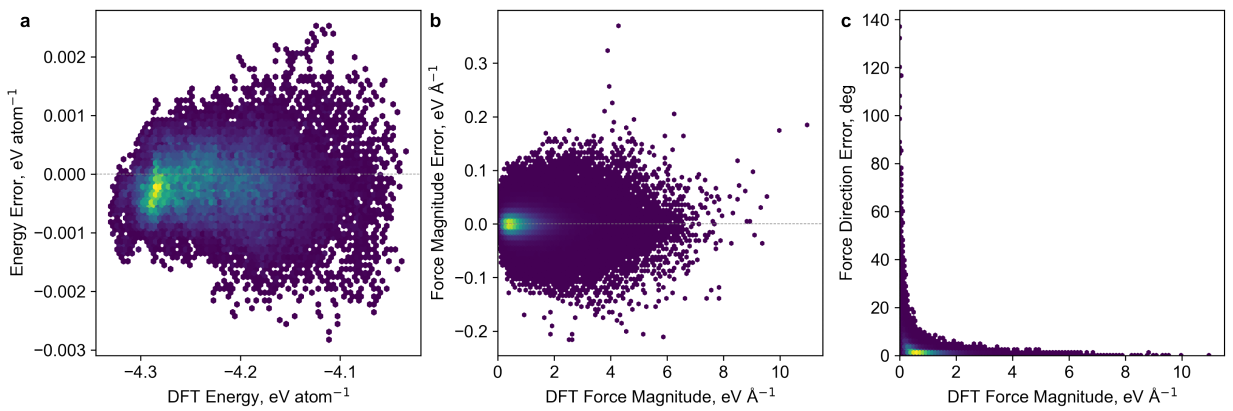

FIG. 3. Errors on (a) energies, (b) force magnitudes, and (c) force directions on the entire collected AIMD dataset. In panels (a) and (b) the horizontal lines represent zero error.

half of the Cl atoms on the 4a Wyckoff sites to create a 50% S/Cl disorder. This is similar to commercially available Li_6PS_5Cl solid electrolytes [11].

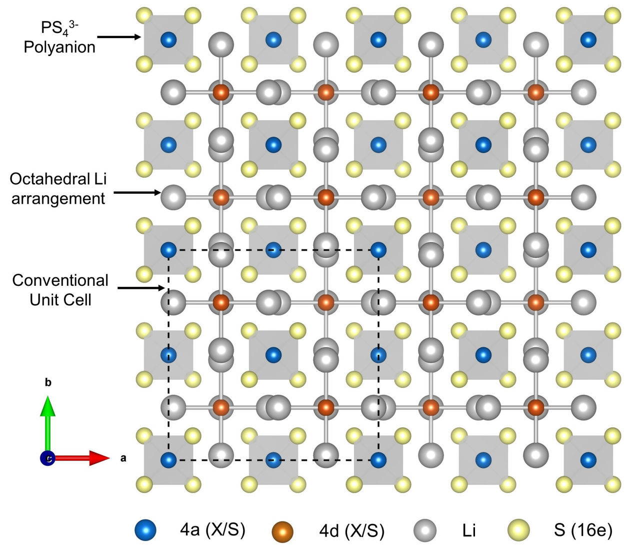

FIG. 4. $2 \times 2 \times 1$ Simulation cell of Li_6PS_5Cl used for the molecular dynamics (MD) simulations in this work, shown projected along the c-axis.

For the sampling of snapshots, we employed isobaric (NPT) simulations using a Nose–Hoover barostat, whereas the velocity autocorrelation function was generated from isovolumetric (NVT) simulations with a Nose–Hoover thermostat. All simulations were performed with an integration timestep of 2 fs. In the NPT simulations, the Nose–Hoover barostat was used with a temperature damping parameter of 10 fs and a pressure damping parameter of

100 fs. The NVT simulations employed the same temperature damping parameter. These values were selected to ensure stable control of pressure and temperature while avoiding overdamping. Prior to production runs, the atomic positions and simulation cell were relaxed in LAMMPS to remove excess potential energy, which would otherwise convert into kinetic energy and destabilize the simulation [12]. To randomise the initial arrangement of lithium ions, an NPT anneal was also performed at 700 K for 10 ps, followed by quenching to the target simulation temperature over 4 ps.

Whilst metadynamics was used to accelerate the sampling of Li-ion diffusion for MLIP training, it was not employed to generate snapshots for NMR tensor averaging. This is because NMR observables must reflect a Boltzmann-weighted occupancy of states. Sampling from biased trajectories would artificially weight high-energy basins and transition states. The sampling of such snapshots would likely overestimate the impact of high-energy Li-ion positions on the NMR tensors. Consequently, all snapshots were selected from unbiased trajectories.

C. Custom Cl Environment Descriptors

To investigate the timescales of Li-ion diffusion shown in Figure 3, we employed two complementary approaches. The first approach characterises the local Li-ion environments surrounding Cl atoms. In the S/Cl substructure, the unit cell is divided into 136 close-packed tetrahedra labelled T0–T5. T0 sites, located at cube edges, are occupied by P atoms, leaving 132 tetrahedra available as potential sites for mobile Li ions. For each Cl atom, we identify the set of nearest-neighbour tetrahedra, including T2 and T4 sites, and generate a Boolean vector, A, indicating whether each site is occupied (Figure 5). This vector provides a physically interpretable descriptor of the local Li-ion environment, which can be compared between different timesteps. To make an ensemble averaged comparison we computed the autocorrelation function:

$$C_{\mathbf{A}}(\Delta) = \frac{\langle \mathbf{A}(t) \cdot \mathbf{A}(t+\Delta) \rangle_t}{\langle \mathbf{A}(t) \cdot \mathbf{A}(t) \rangle_t}, \tag{1}$$

where $\mathbf{A}(t)$ is the descriptor at time t, Δ is the time lag, $\langle \cdot \rangle_t$ denotes a time average over all t. The dot product, $\mathbf{A}(t) \cdot \mathbf{A}(t+\Delta)$, quantifies the correlation between the vector at time t and its value at a later time $t + \Delta$.

The second approach analyses Li-ion dynamics by computing the radially integrated part of the self van Hove autocorrelation function, $G_s(r,t)$, defined as

$$G_s(r,t) = \frac{1}{N}\sum_{i=1}^{N} \langle \delta \left(r - |\mathbf{r}_i(t+\Delta) - \mathbf{r}_i(t)| \right) \rangle_t \,, \tag{2}$$

where $\mathbf{r}_i(t)$ is the position of the i-th Li ion at time t and N is the number of mobile (Li) ions. $G_s(r,t)$ represents the probability of finding a Li-ion within a distance r of its initial position after a time interval Δ. Integrating over r enables quantification of the displacement timescales, providing a complementary measure of diffusive behaviour consistent with the cage-based descriptor.

D. Calculation of NMR tensors

Geometry optimisations and first principles NMR calculations were performed using the plane-wave DFT code CASTEP [13]. A maximum planewave energy of 900 eV and k-point spacing of 0.05 Å^{-1} were used for the calculations, which was found to produce δ_{iso} values converged to within ± 0.01 ppm. The PBE functional [14] was used for all calculations. Magnetic shielding, σ, and electric field gradient (EFG), V, tensors were calculated using the GIPAW method [15–17]. Although magnetic shieldings are not directly measured, they relate to the experimental chemical shift δ via a reference shielding σ_{ref}:

$$\delta = \sigma_{\text{ref}} - \sigma \tag{3}$$

To determine σ_{ref}, a linear regression is performed between experimental isotropic shifts δ_{iso} and calculated isotropic shieldings σ_{iso}, defined as

$$\sigma_{\text{iso}} = \tfrac{1}{3}\left(\sigma_{11} + \sigma_{22} + \sigma_{33}\right), \tag{4}$$

where σ_{ii} are the principal components of the symmetric part of the shielding tensor $\sigma_{\text{sym}} = \frac{1}{2}\left(\sigma + \sigma^T\right)$. The intercept of this regression yields σ_{ref}. While the regression slope is often allowed to deviate from –1 to improve agreement with experiment, here we fix it at –1 to isolate systematic errors arising from motional effects. In this work we obtain a value of 939.29 ppm for σ_{ref} by performing this procedure against a series of alkali chlorides, as shown in Figure 6.

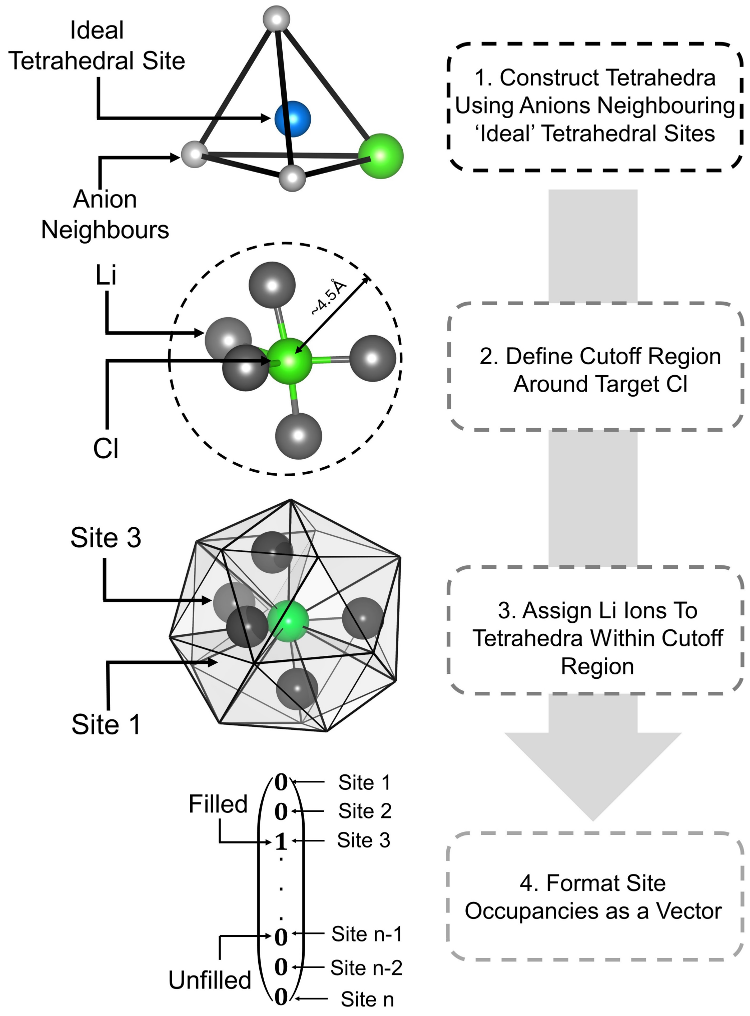

FIG. 5. Schematic illustrating the construction of the custom Cl environment descriptors. For each Cl atom, the neighbouring close-packed tetrahedra are identified, and a Boolean vector is generated to indicate whether each tetrahedron is occupied by a Li ion.

Several useful parameters can be derived from the NMR tensors. First from the shielding tensor we define the isotropic chemical shift, δ_{iso}, and reduced shift anisotropy, δ_{aniso} as follows:

$$\delta_{\text{iso}} = \frac{1}{3}\left(\delta_{11} + \delta_{22} + \delta_{33}\right). \tag{5}$$

$$\delta_{\text{aniso}} = \delta_{33} - \delta_{\text{iso}}. \tag{6}$$

The Haeberlen convention [19] has been used in this work:

$$|\delta_{33} - \delta_{\text{iso}}| \geq |\delta_{11} - \delta_{\text{iso}}| \geq |\delta_{22} - \delta_{\text{iso}}|. \tag{7}$$

δ_{ii} are the principal components of the symmetric part of the shift tensor $\delta_{\text{sym}} = \frac{1}{2}\left(\delta + \delta^{T}\right)$.

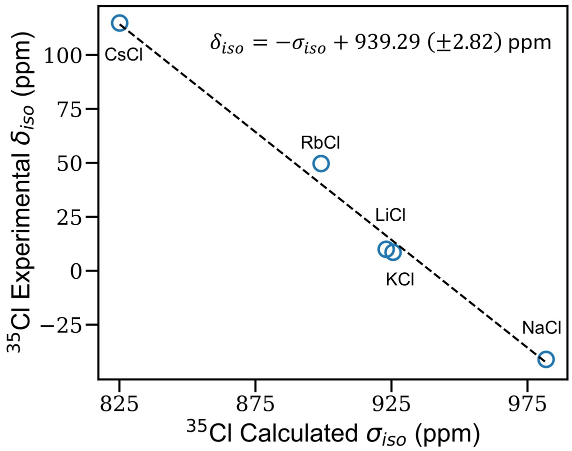

FIG. 6. Calculation of σ_{ref} from a linear regression performed between a set of experimental δ_{iso} and calculated σ_{iso} values from Ref. 18

The isotropic chemical shift, δ_{iso}, describes the magnitude of the shift tensor whereas δ_{aniso} has been used in this work as a description of the anisotropy of the shift tensor.

For the EFG tensor we have derived the quadrupolar coupling constant, C_Q, and asymmetry parameter, η_Q:

$$C_Q = \frac{eQV_{zz}}{h}. \tag{8}$$

$$\eta_Q = \frac{V_{xx} - V_{yy}}{V_{zz}} \tag{9}$$

where h is Planck's constant, eQ is the electric quadrupole moment and the following convention has been used:

$$|V_{zz}| \geq |V_{yy}| \geq |V_{xx}|. \tag{10}$$

The quadrupolar coupling constant, C_Q, quantifies the magnitude of the largest principal component of the electric field gradient. In this work, C_Q, has been used to characterise the anisotropy of the EFG tensor as it relates to the uniformity of the electric field gradient. The asymmetry parameter, η_Q, has also been employed to characterise shape as it describes the deviation of the EFG tensor from axial symmetry.

Finally, for both tensors we define tensor reorientation angles β by projecting the eigenvectors of the tensors at different timesteps onto a reference orientation frame. We note for the computation of the eigenvectors of the shift tensor, δ, the symmetric part of the tensor has been used, $\delta_{\text{sym}} = \frac{1}{2}\left(\delta + \delta^T\right)$.

E. Experimental NMR

The Li_6PS_5Cl powder was obtained from Ampcera and used as received. All samples were packed in argon gloveboxes (less than 1 ppm H_2O and O_2) and the experiments were completed under dry nitrogen conditions.

The ^{35}Cl NMR measurements in Figure 5 were performed at 20 T (ν_0= 83.30 MHz) using a Bruker NEO spectrometer. In addition, to derive an experimental estimate for C_Q a second dataset was obtained at 9.45 T (ν_0= 39.21 MHz) using a Bruker Avance III HD spectrometer. At 9.45 T, a Bruker 4 mm low-gamma probe was employed, with data acquired under both static and magic-angle spinning (MAS, ν_R=12 kHz) conditions.

At 20 T, a 7 mm laser probe was used to enable sample heating up to 300 °C, providing both static and MAS (ν_R= 4.2 kHz) measurements. A relaxation delay of 0.5 s was applied at both fields, with a minimum of 1024 scans collected for each experiment. All experiments were referenced to Na^{35}Cl(s) at −46.1 ppm [20, 21].

Using the datasets obtained at two different magnetic field (9.45 T, 20 T) we have obtained an isotropic chemical shift using the following relationship [22]:

$$\delta_{iso} = \frac{\nu_1^2 \delta_{cg1} - \nu_2^2 \delta_{cg2}}{\nu_1^2 - \nu_2^2} \tag{11}$$

where δ_{cg1} and δ_{cg2} are the centre of gravity shifts, corresponding to the positions of the solid-state peaks, and ν_1 and ν_2 are the Larmor frequencies at the two different field strengths.

A value for C_Q was also estimated from the experimentally derived quadrupole coupling parameter, P_Q, which is related to δ_{iso} through:

$$\delta_{cg} = \delta_{\text{iso}} - \frac{3}{500} \frac{P_Q^2}{\nu^2} \times 10^6, \tag{12}$$

where δ_{cg} is the centre of gravity of the resonance, and ν is the Larmor frequency. The quadrupolar coupling constant C_Q is then related to P_Q by:

$$P_Q = C_Q \sqrt{1 + \frac{\eta_Q^2}{3}} \approx C_Q, \tag{13}$$

where the approximation holds for small asymmetry parameters ($\eta_Q \ll 1$).

F. Simulating NMR Spectra

Parameter	Value
Magnetic Field (T)	19.96
Larmor Frequency (MHz)	83.3
Sample Spinning (kHz)	0
RF-Field (kHz)	125
Pulse Length (μs)	2
Broadening (MHz)	1749
Crystal File	zcw4180
Gamma Angles	1

TABLE I. SIMPSON simulation parameters.

The calculated NMR parameters were used to simulate spectra via the SIMPSON program [23]. In short this package independently solves the time-dependent spin Hamiltonian of the individual nuclei. The input parameters of the simulation were chosen to match those used in the 20 T experimental NMR simulation and are given in Table I. Additional parameters such as the spectral broadening, the crystal file and gamma angles were chosen to create a converged spectrum. For each model, the NMR spectrum of each nucleus was simulated independently, and then summed together to make the final NMR spectra for each element. We have also normalised the intensity of the simulated spectra to match the peak with that of the experimental spectra.

RESULTS

G. Validation of our MLIP

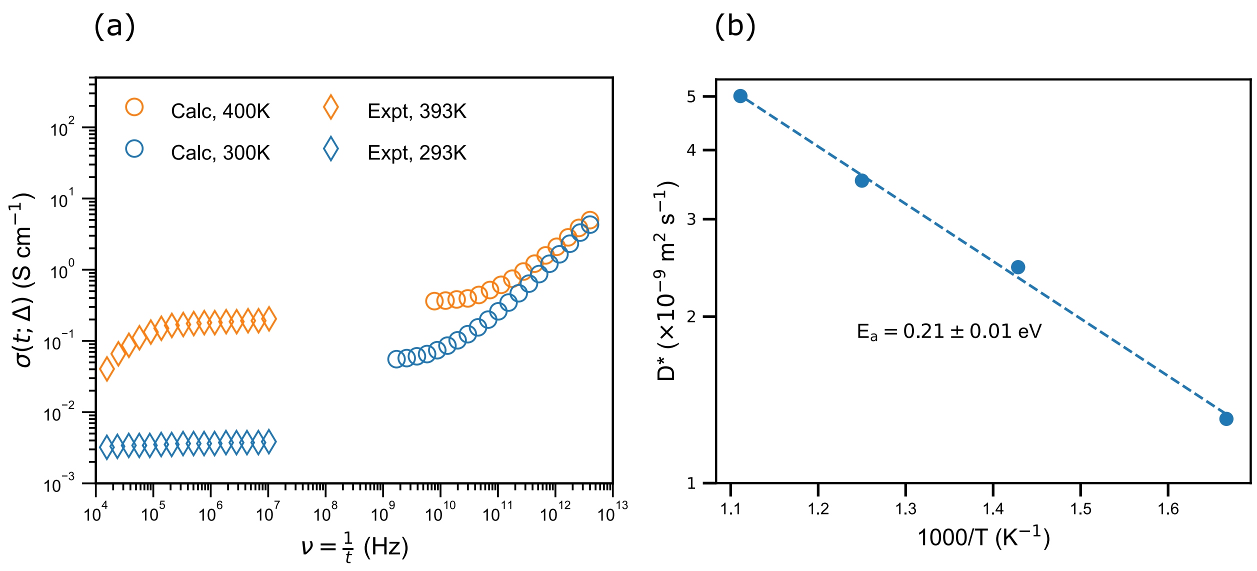

FIG. 7. (a) Comparison of the calculated (Calc) AC ionic conductivities and a set of experimental (Expt) AC ionic conductivities. (b) The Arrhenius plot of the tracer diffusivity, D*, obtained from the MSDs.

Since our method focuses on sampling configurations representative of Li-ion motion, we have centred our validation on the accuracy with which Li diffusion is captured by our MLIP. This was assessed in two ways. Following the approach of Poletayev et al. [24], we first computed the frequency-dependent ionic conductivities, $\sigma(\nu)$. The conductivity $\sigma(t)$ sampled over a time interval t is related to the elementary charge q, Boltzmann's constant k_B, the temperature T, and the number of Li ions N within the simulation volume V via the following equation:

$$\sigma(t) = \frac{1}{V}\frac{(qN)^2}{k_B T} D_{\mathrm{CoM}}(t) \tag{14}$$

where D_{CoM} is the centre-of-mass diffusion coefficient. This diffusion coefficient is related to the autocorrelation function of the squared centre-of-mass displacement:

$$D_{\mathrm{CoM}} = \frac{\langle r^2_{\mathrm{CoM}}(0)\ r^2_{\mathrm{CoM}}(t)\rangle}{6t} \tag{15}$$

which we have computed from our NPT simulations.

Figure S7a compares the resulting conductivities with experimental AC conductivity values obtained by Hanghofer et al. [25] using impedance spectroscopy. The frequency dependence of ionic conductivity is typically described by a Jonscher-type power law:

$$\sigma(\nu) = \sigma_{DC} + A\nu^p \tag{16}$$

where A and p are constants, and σ_{DC} is the DC plateau commonly observed in the low-frequency regime. This is also the regime probed by macroscopic experiments. Due to the limited timescale accessible in our simulations, the conductivities extracted from the MLIP trajectories correspond to the high-frequency regime. The accuracy of the MLIP is therefore evaluated by examining how well the calculated conductivities extrapolate towards the DC plateau. Given the uncertainties associated with defects in real samples, the agreement between the experimental DC plateau and our pristine simulations is encouraging.

To further validate the accuracy of our potential at describing Li-ion diffusion we have computed tracer diffusivities, $D*$, using the following relationship:

$$D^* = \frac{1}{6N}\sum_{i=1}^{N}\frac{r_i(t)^2}{t} \tag{17}$$

where $r_i(t)^2$ is the mean squared displacement of the Li ions sampled over a time t. For this computation, we have ensured that the sampling times used are long enough for Fickian diffusion to be observed. We repeated this analysis at several temperatures to obtain the Arrhenius plot in Figure 7b from which we obtain an activation energy, E_a, of 0.21 ± 0.01 eV. This result is in good agreement with previous AIMD calculations (0.18 ± 0.02 eV[26]). Hanghofer et al. reported a higher value of 0.396 ± 0.003 eV[25] based on impedance spectroscopy measurements. The discrepancy (over-estimation of room-temperature ionic conductivity) is likely due to the finite size of the simulation cells used in our calculations, which are constrained by the cost of DFT used subsequently for computing electric field gradients, the influence of defects, and possible non-Arrhenius behaviour between the temperatures simulated in Figure 7b and room temperature: as we show below, the material appears to change above 250 °C (Figure 9). Considering these limitations, the agreement between our results and literature experimental and computational data is satisfactory.

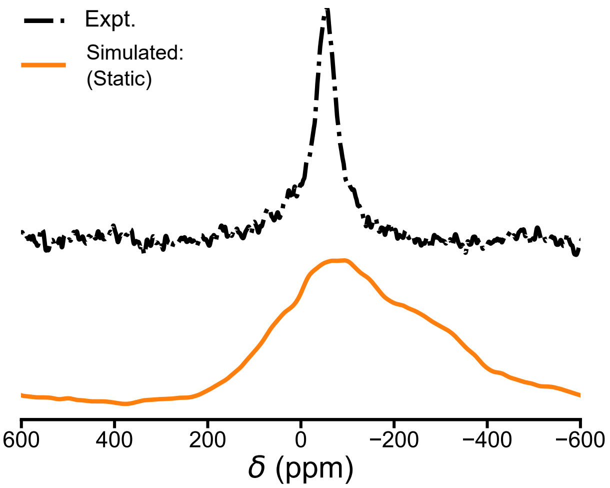

FIG. 8. Comparison of the experimental static ^{35}Cl NMR spectrum of Li_6PS_5Cl with the spectrum simulated using the conventional single–static-structure approach.

H. Static NMR spectra

Figure 8 compares the experimental ^{35}Cl NMR spectrum of Li_6PS_5Cl with that obtained using the conventional approach. For this comparison, NMR parameters from a single snapshot of our simulation were used to generate the spectrum. As shown in Figure 8, the conventional approach, which relies on a single relaxed static structure, significantly overestimates the width of the experimental peak. This overestimation limits the ability of a method based on a static structure to capture subtle changes in peak shape or position, reducing its effectiveness for interpreting variations in the underlying atomic structure.

I. Temperature-dependent NMR spectra

A series of NMR experiments were carried out with heating the Li_6PS_5Cl argyrodite material to determine the origin of the narrow peak at 7.2 ppm. This peak was absent in the as-synthesized argyrodite material but appeared upon heating above 250 °C, and remained present upon cooling (Figure 9). This experiment suggests that the peak at 7.2 ppm is not due to an intrinsic component of the argyrodite material itself, but is consistent with thermal decomposition to LiCl. Similar work mixed-halide argyrodites also detects an analogous mixed-halide rocksalt impurity [27].

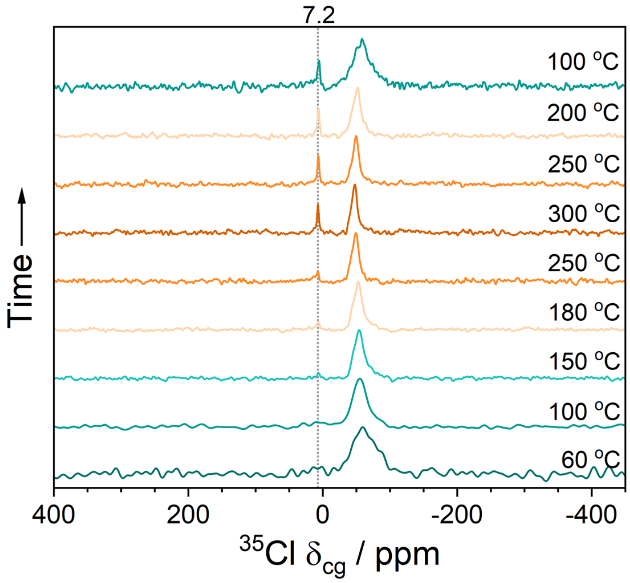

FIG. 9. ^{35}Cl MAS NMR spectra collected over the course of heating the Li_6PS_5Cl material from room temperature to 300 C and cooling to room temperature.

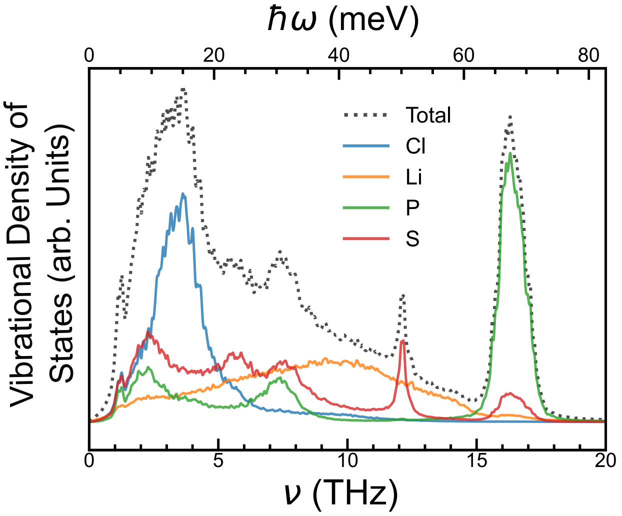

FIG. 10. Vibrational density of states of Li_6PS_5Cl obtained from MD simulations in the isovolumetric (NVT) ensemble at 300 K using our custom MLIP.

J. Velocity autocorrelation function (VACF)

The velocity autocorrelation function (VACF) was computed as:

$$C_v(t) = \frac{\langle \mathbf{v}_i(t) \cdot \mathbf{v}_i(t+\Delta) \rangle}{\langle \mathbf{v}_i(t) \cdot \mathbf{v}_i(t) \rangle}, \tag{18}$$

where $\mathbf{v}_i(t)$ is the velocity of particle i at time t, and $\langle \cdots \rangle$ denotes an ensemble average over all particles and time lags, Δ.

The VACF of species s is related to its partial density of states (pDoS), $g_s(\omega)$, via the Fourier transform

$$g_s(\omega) = \int_{\infty}^{\infty} e^{j\omega t} \frac{\langle \mathbf{v}_i(t) \cdot \mathbf{v}_i(t+\Delta) \rangle_s}{\langle \mathbf{v}_i(t) \cdot \mathbf{v}_i(t) \rangle_s} dt \tag{19}$$

where j denotes $\sqrt{-1}$ and $\langle \cdots \rangle_s$ denotes the computation of the autocorrelation function only over particles of species s. The total phonon, or vibrational, density of states (vDoS), $g(\omega)$ is obtained by summing over all species:

$$g(\omega) = \sum_s \int_{\infty}^{\infty} e^{j\omega t} \frac{\langle \mathbf{v}_i(t) \cdot \mathbf{v}_i(t+\Delta) \rangle_s}{\langle \mathbf{v}_i(t) \cdot \mathbf{v}_i(t) \rangle_s} dt \tag{20}$$

We have computed both quantities in Figure 10 using NVT simulations run at 300 K. The VACF of Cl exhibits a distinct peak at 5 THz and decays to zero around 10 THz, indicating that the majority of Cl vibrational modes are concentrated below 10 THz. In contrast, the VACF of Li shows a broader peak around 10 THz and decays by 20 THz, reflecting faster average Li-ion vibrations. These higher-frequency Li vibrations are expected to contribute more significantly to the vibrational averaging of the NMR parameters.

[1] G. Kresse and J. Hafner, Ab initio molecular-dynamics simulation of the liquid-metal–amorphous-semiconductor transition in germanium, Physical Review B **49**, 14251 (1994).

[2] G. Kresse and J. Furthmüller, Efficiency of ab-initio total energy calculations for metals and semiconductors using a plane-wave basis set, Computational Materials Science **6**, 15 (1996).

[3] J. P. Perdew, A. Ruzsinszky, G. I. Csonka, O. A. Vydrov, G. E. Scuseria, L. A. Constantin, X. Zhou, and K. Burke, Restoring the density-gradient expansion for exchange in solids and surfaces, Phys. Rev. Lett. **100**, 136406 (2008).

[4] S. Batzner, A. Musaelian, L. Sun, M. Geiger, J. P. Mailoa, M. Kornbluth, N. Molinari, T. E. Smidt, and B. Kozinsky, E(3)-equivariant graph neural networks for data-efficient and accurate interatomic potentials, Nature Communications **13**, 2453 (2022).

[5] A. Musaelian, S. Batzner, A. Johansson, L. Sun, C. J. Owen, M. Kornbluth, and B. Kozinsky, Learning local equivariant representations for large-scale atomistic dynamics, Nature Communications **14**, 579 (2023).

[6] M. Geiger and T. Smidt, e3nn: Euclidean neural networks (2022), arXiv:2207.09453 [cs.LG].

[7] D. P. Kingma and J. Ba, Adam: A Method for Stochastic Optimization, in *International Conference on Learning and Representation* (2015).

[8] A. D. Poletayev, Training data and models for molecular dynamics of Li_6PS_5Cl, 10.5281/zenodo.18902498 (2026).

[9] T. Maxson, A. Soyemi, B. W. J. Chen, and T. Szilvási, Enhancing the Quality and Reliability of Machine Learning Interatomic Potentials through Better Reporting Practices, The Journal of Physical Chemistry C **128**, 6524 (2024).

[10] A. P. Thompson, H. M. Aktulga, R. Berger, D. S. Bolintineanu, W. M. Brown, P. S. Crozier, P. J. in 't Veld, A. Kohlmeyer, S. G. Moore, T. D. Nguyen, R. Shan, M. J. Stevens, J. Tranchida, C. Trott, and S. J. Plimpton, Lammps - a flexible simulation tool for particle-based materials modeling at the atomic, meso, and continuum scales, Computer Physics Communications **271**, 108171 (2022).

[11] B. T. Payne, M. Juelsholt, M. A. Pérez-Osorio, D. L. R. Melvin, G. J. Cuello, E. Suard, D. J. M. Irving, N. H. Rees, M. Feaviour, E. Petrucco, S. P. Day, G. J. Rees, and P. G.

Bruce, How multi-length scale disorder shapes ion transport in lithium argyrodites, Energy & Environmental Science **18**, 8876 (2025).

[12] M. Dračínský and P. Bouř, Vibrational averaging of the chemical shift in crystalline α-glycine, Journal of Computational Chemistry **33**, 1080 (2012), https://onlinelibrary.wiley.com/doi/pdf/10.1002/jcc.22940.

[13] S. J. Clark, M. D. Segall, C. J. Pickard, P. J. Hasnip, M. I. J. Probert, K. Refson, and M. C. Payne, First principles methods using castep, Zeitschrift für Kristallographie - Crystalline Materials **220**, 567 (2005).

[14] J. P. Perdew, K. Burke, and M. Ernzerhof, Generalized gradient approximation made simple, Physical review letters **77**, 3865 (1996).

[15] C. J. Pickard and F. Mauri, All-electron magnetic response with pseudopotentials: NMR chemical shifts, Physical Review B **63**, 245101 (2001).

[16] J. R. Yates, C. J. Pickard, and F. Mauri, Calculation of nmr chemical shifts for extended systems using ultrasoft pseudopotentials, Phys. Rev. B **76**, 024401 (2007).

[17] C. Bonhomme, C. Gervais, F. Babonneau, C. Coelho, F. Pourpoint, T. Azaïs, S. E. Ashbrook, J. M. Griffin, J. R. Yates, F. Mauri, and C. J. Pickard, First-principles calculation of nmr parameters using the gauge including projector augmented wave method: A chemist's point of view, Chem. Rev. **112**, 5733 (2012), https://doi.org/10.1021/cr300108a.

[18] R. P. Chapman, C. M. Widdifield, and D. L. Bryce, Solid-state NMR of quadrupolar halogen nuclei, Progress in Nuclear Magnetic Resonance Spectroscopy **55**, 215 (2009).

[19] U. Haeberlen, *High Resolution NMR in Solids: Selective Averaging*, Advances in Magnetic Resonance. Supplement No. 1 (Academic Press, New York, 1976).

[20] K. J. D. MacKenzie and M. E. Smith, *Multinuclear solid-state nuclear magnetic resonance of inorganic materials* (Elsevier, 2002).

[21] H. Zhang and I. Farnan, High temperature 35cl nuclear magnetic resonance study of the licl–kcl system and the effect of cecl3 dissolution, Faraday Discussions **190**, 367 (2016).

[22] G. J. Rees, S. P. Day, A. Lari, A. P. Howes, D. Iuga, M. B. Pitak, S. J. Coles, T. L. Threlfall, M. E. Light, M. E. Smith, D. Quigley, J. D. Wallis, and J. V. Hanna, A multinuclear solid state nmr, density functional theory and x-ray diffraction study of hydrogen bonding in group i hydrogen dibenzoates, CrystEngComm **15**, 8823 (2013).

[23] M. Bak, J. T. Rasmussen, and N. C. Nielsen, SIMPSON: A General Simulation Program for

Solid-State NMR Spectroscopy, Journal of Magnetic Resonance **147**, 296 (2000).

[24] A. D. Poletayev, J. A. Dawson, M. S. Islam, and A. M. Lindenberg, Defect-driven anomalous transport in fast-ion conducting solid electrolytes, Nature Materials **21**, 1066 (2022).

[25] I. Hanghofer, M. Brinek, S. L. Eisbacher, B. Bitschnau, M. Volck, V. Hennige, I. Hanzu, D. Rettenwander, and H. M. R. Wilkening, Substitutional disorder: structure and ion dynamics of the argyrodites Li 6 PS 5 Cl, Li 6 PS 5 Br and Li 6 PS 5 I, Physical Chemistry Chemical Physics **21**, 8489 (2019).

[26] N. J. De Klerk, I. Rosłoń, and M. Wagemaker, Diffusion Mechanism of Li Argyrodite Solid Electrolytes for Li-Ion Batteries and Prediction of Optimized Halogen Doping: The Effect of Li Vacancies, Halogens, and Halogen Disorder, Chemistry of Materials **28**, 7955 (2016).

[27] P. Wang, S. Patel, H. Liu, P. Chien, X. Feng, L. Gao, B. Chen, J. Liu, and Y.-Y. Hu, Configurational and Dynamical Heterogeneity in Superionic Li 5.3 PS 4.3 Cl 1.7 x Br x, Advanced Functional Materials **33**, 1 (2023).
